# Editorial: Understanding Early Detection Markers in Schizophrenia

**DOI:** 10.3389/fnbeh.2021.724509

**Published:** 2021-07-16

**Authors:** Stella G. Giakoumaki, Kyriaki Sidiropoulou, Antonis Stamatakis

**Affiliations:** ^1^Laboratory of Neuropsychology, Department of Psychology, Faculty of Social Sciences, University of Crete, Rethymno, Greece; ^2^University of Crete Research Center for the Humanities, The Social and Educational Sciences, University of Crete, Rethymno, Greece; ^3^Department of Biology, University of Crete, Heraklion, Greece; ^4^Institute of Molecular Biology and Biotechnology-Foundation for Research and Technology Hellas, Heraklion, Greece; ^5^Biology-Biochemistry Lab, Faculty of Nursing, School of Health Sciences, NKUA, Athens, Greece

**Keywords:** Heschl's gyrus, cell-free DNA, occulomotor function, schizotypy, first-episode antipsychotic-naive schizophrenia, childhood trauma, cognitive symptoms

Schizophrenia is a chronic debilitating disorder that affects 1% of the population. It refers to a heterogeneous set of symptoms including psychosis, cognitive deficits, social and functional impairments, and typically emerges in young adulthood. The lack of firm knowledge with regards to the etiology of schizophrenia despite the longstanding research in the field, the difficulty in diagnosis due to the associated stigma and the fact that several indices (cognitive, neurobiological, neuroanatomical) are already impaired prior to diagnosis in individuals at increased genetic or clinical risk for the disorder, hinder early treatment interventions. As the study of schizophrenic processes in patients has important methodological limitations (e.g., the effects of illness chronicity), the study of high-risk populations can provide valuable information on the early mechanisms associated with the disorder. Additionally, the use of animal models can shed light on developmental mechanisms involved in the pathogenesis of schizophrenia. This special Research Topic in Frontiers in Psychiatry and Frontiers in Behavioral Neuroscience brings together different studies which provide evidence for or against specific biomarkers for the detection of and susceptibility to schizophrenia. The main findings of the included studies are summarized below.

## Molecular Markers for Schizophrenia

In the past decade, significant progress has been made toward identifying high-risk polymorphisms associated with schizophrenia. However, these data so far have not been translated to identifiable biomarkers for the disease. In one of the studies in this topic, the case is made that these susceptibility polymorphisms could be important in specific sub-populations with a family history of schizophrenia and/or exposure to childhood trauma. Specifically, the authors showed that NEDD4 single-nucleotide polymorphisms and childhood trauma are associated with increased morbidity, especially in people with a family history of psychoses (Bi et al.). In an additional effort to identify molecular biomarkers for schizophrenia, Li et al. investigated the implication of various types of RNAs (mRNAs, miRNAs, and lncRNAs) in schizophrenia. They identified differential **expression of autophagy-related RNAs possibly** comprising a ceRNA network. Moreover, certain miRNAs showed a potential predictive value for the diagnosis of the disease (Li et al.). Finally, the validity for using serum cell-free DNA levels as a biomarker for schizophrenia was examined by Chen et al. The study reports that schizophrenia patients exhibit increased levels of serum cfDNA compared to healthy controls, which is reduced by antipsychotics, further reinforcing the idea that cfDNA levels can be used as an additional diagnostic marker in schizophrenia. However, no predictive value of cfDNA levels for dissociating different types of schizophrenia and the age of onset among others was identified (Chen et al.).

## Anatomical Markers

In a structural imaging study, Takahashi et al. examined the gyrification pattern of Heschl's gyrus (HG) in at-risk mental state (ARMS) individuals, schizophrenia patients and a group of healthy controls and further assessed differences in clinical variables of participants grouped according to their gyrification patterns. They found that (a) the prevalence of duplicated HG patterns bilaterally was higher in the ARMS and schizophrenia groups and (b) the left common stem duplication pattern was associated with verbal fluency, mainly in the schizophrenia group, and with general psychopathology in the ARMS group (Takahashi et al.). These findings provide further evidence in the literature reporting altered structural brain patterns as vulnerability indicators to schizophrenia.

## Behavioral Markers/Endophenotypes

Peng et al. assessed the relationship between clinical characteristics and cognitive deficits in **first-episode drug-naïve schizophrenia** patients. They detected a higher prevalence of cognitive deficits among the patients compared to the general population. Moreover, they identified a negative correlation of negative symptoms with cognitive functioning as well as of general pathology with attention/vigilance. No correlation was found between cognitive deficits and positive symptoms (Peng et al.). These findings corroborate the idea of utilizing certain types of clinical symptoms as predictors of cognitive deficits in schizophrenia patients. Karamaouna et al. carried out a follow-up study involving assessments of schizotypal traits and cognition at two time points (baseline and 4 years after the baseline assessment). They reported (a) differential stability of cognitive functions and schizotypal traits over time depending on the dimension of schizotypy, (b) that negative and disorganized baseline schizotypal traits are associated with a different pattern of cognitive functioning after 4 years of the initial assessment, and (c) that a set of cognitive functions are stably impaired in high negative schizotypal individuals while another set might be more state-dependent (Karamaouna et al.). Their findings are significant contributors in the formulation of targeted early-intervention strategies for high-risk populations.

In a related subject, Athanasopoulos et al. reviewed studies involving the use of oculomotor function indices as diagnostic, susceptibility, predictive, monitoring, and prognostic biomarkers for schizophrenia. After a thorough revision of the literature, they concluded that the existing literature cannot sufficiently support the use of oculomotor function as a diagnostic biomarker but they propose that oculomotor function can be used as a monitoring biomarker, especially as new therapies for cognitive symptoms become available (Athanasopoulos et al.).

## Animal Studies

Animal studies are also critical for understanding the neuronal mechanisms involved in the emergence of schizophrenia. Understanding adaptations in synaptic transmission players, such as the NMDA receptor, at different developmental time points will further enable the delineation of the mechanisms involved in the manifestation of different symptoms. In this topic, Plataki et al. showed that NMDA receptor hypofunction at distinct early life postnatal periods can differentially affect the function of the prefrontal cortex and the hippocampus. Specifically, the function of the hippocampus is altered only when NMDA receptor hypofunction occurs after the first postnatal week in the mouse, while prefrontal cortical function is affected when NMDA receptors are blocked in the second half of the second postnatal week (Plataki et al.).

## Conclusion

The articles in this topic highlight the complexity in understanding the full spectrum of schizophrenia-related symptoms and adaptations. The human studies further validate the idea that cognitive impairment is a core feature of schizophrenia and related disorders while the animal study in this topic reveals the significant role of early postnatal development in the disruption of cognitive function.

## Author Contributions

SG, KS, and AS wrote the editorial. All authors contributed to the article and approved the submitted version.

## Conflict of Interest

The authors declare that the research was conducted in the absence of any commercial or financial relationships that could be construed as a potential conflict of interest.

